# In-Hospital Mortality Prediction among Intensive Care Unit Patients with Acute Ischemic Stroke: A Machine Learning Approach

**DOI:** 10.34133/hds.0179

**Published:** 2025-03-17

**Authors:** Jack A. Cummins, Ben S. Gerber, Mayuko Ito Fukunaga, Nils Henninger, Catarina I. Kiefe, Feifan Liu

**Affiliations:** ^1^ Manchester Essex Regional High School, Manchester, MA 01944, USA.; ^2^ Department of Population and Quantitative Health Sciences, UMass Chan, Worcester, MA 01665, USA.; ^3^ Division of Health Informatics and Implementation Science, UMass Chan, Worcester, MA 01655, USA.; ^4^ Division of Pulmonary, Allergy and Critical Care Medicine, UMass Chan, Worcester, MA 01655, USA.; ^5^ Meyers Primary Care Institute, Worcester, MA 01605, USA.; ^6^ Department of Neurology, UMass Chan, Worcester, MA 01655, USA.

## Abstract

**Background:** Acute ischemic stroke is a leading cause of death in the United States. Identifying patients with stroke at high risk of mortality is crucial for timely intervention and optimal resource allocation. This study aims to develop and validate machine learning-based models to predict in-hospital mortality risk for intensive care unit (ICU) patients with acute ischemic stroke and identify important associated factors. **Methods:** Our data include 3,489 acute ischemic stroke admissions to the ICU for patients not discharged or dead within 48 h from the Medical Information Mart for Intensive Care-IV (MIMIC-IV) database. Demographic, hospitalization type, procedure, medication, intake (intravenous and oral), laboratory, vital signs, and clinical assessment [e.g., Glasgow Coma Scale Scores (GCS)] during the initial 48 h of admissions were used to predict in-hospital mortality after 48 h of ICU admission. We explored 3 machine learning models (random forests, logistic regression, and XGBoost) and applied Bayesian optimization for hyperparameter tuning. Important features were identified using learned coefficients. **Results:** Experiments show that XGBoost tuned for area under the receiver operating characteristic curve (AUC ROC) was the best performing model (AUC ROC 0.86, F1 0.52), compared to random forests (AUC ROC 0.85, F1 0.47) and logistic regression (AUC ROC 0.75, F1 0.40). Top features include GCS, blood urea nitrogen, and Richmond RASS score. The model also demonstrates good fairness for males versus females and across racial/ethnic groups. **Conclusions:** Machine learning has shown great potential in predicting in-hospital mortality risk for people with acute ischemic stroke in the ICU setting. However, more ethical considerations need to be applied to ensure that performance differences across different racial/ethnic groups will not exacerbate existing health disparities and will not harm historically marginalized populations.

## Introduction

Acute stroke often requires intensive care management and is associated with high mortality [1]. Identifying patients at high risk of acute stroke-related mortality can help healthcare teams prioritize resources and improve clinical decision-making (e.g., intensifying monitoring or withdrawing from maximum therapy) [2,3]. A combination of physical exam and electronic health record findings serves as a resource for risk prediction efforts by clinicians. For example, a number of prognostic scores have been developed to assess the overall condition severity or mortality risk, such as the Oxford Acute Severity of Illness Score (OASIS) [4], the Glasgow Coma Scale (GCS) [5], and the National Institutes of Health Stroke Scale (NIHSS) [6]. Neurologic evaluation of patients’ cognition, motor skills, sensation, and verbal response combined with past history (e.g., atrial fibrillation, diabetes, prior stroke, and hypertension), vital signs (e.g., heart rate, blood pressure, and temperature), laboratory studies [e.g., glucose, creatinine, blood urea nitrogen (BUN), blood counts, and electrolytes], as well as age, is largely known to guide clinician management and predict short-term mortality. Despite their wide availability, the ability of prognostic scores to predict in-hospital mortality is imperfect [7]. For instance, the GCS, OASIS, and NIHSS instruments use a limited number of variables (3, 10, and 15, respectively) and generally only identify linear and well-established relationships. To illustrate a nonlinear relationship, one retrospective cohort study found a nonlinear negative relationship between platelet count and in-hospital mortality among intensive care unit (ICU) patients with stroke [8].

To address these limitations, researchers have increasingly explored machine learning (ML) techniques for clinical risk prediction. They shift the paradigm from simple additive models to more sophisticated algorithms that capture both linear and nonlinear relations. Gradient boosting tree models, such as XGBoost, LightGBM, and CatBoost, have demonstrated higher levels of performance compared with other traditional ML models, such as random forest and logistic regression [9,10].

Previous studies have created ML-based models to predict stroke-related in-hospital mortality [11]. A number use either the Medical Information Mart for Intensive Care-III (MIMIC-III) or MIMIC-IV dataset containing critical care electronic health record data on patients hospitalized in Boston, MA [12]. One study found an XGBoost algorithm effective at predicting in-hospital mortality specifically in patients with intracerebral hemorrhage and is currently available as a web-based calculator. A second study also found XGBoost to be accurate in predicting 28-d in-hospital mortality among hypertensive ischemic or hemorrhagic stroke patients admitted to the ICU [13]. Using SHAP (SHapley Additive exPlanations) values, a number of predictive features were identified for interpretability, such as glucose, age, peripheral oxygen saturation, white blood cell count, and ethnicity. Similarly, other models [14] have been developed with additional datasets (e.g., eICU dataset [15], a large multi-center critical care database from Phillips Healthcare containing EHR (electronic health record) data from admissions at 208 hospitals; National Multiethnic Stroke Registry) that also show promise of XGBoost models for elderly patients with ischemic stroke as well as accurate prediction of longer-term mortality (i.e., 1 year after stroke).

Although the reported performance of these models is promising, there are several limitations among those studies, including a limited number of variables or features and a combined mixture of stroke types (acute/chronic or ischemic/hemorrhagic). In addition, prior studies lack a dynamic representation of longitudinal clinical data; instead, “sliding windows” would allow for better capturing temporal dependencies within longitudinal data (e.g., vital signs and laboratory tests) and thus richer data representations for accurate prediction. More importantly, no studies assessed model fairness across different demographic groups. Previous studies outside of the MIMIC-IV dataset have found that stroke mortality prediction models often have lower performance for non-white individuals than for white individuals [16]. This has been an important ethical consideration hindering the rapid adoption of existing prediction models in clinical practice.

The goal of this study was to develop a reliable, comprehensive, and fair ML model for ICU in-hospital mortality prediction among people with acute ischemic stroke. We specifically focused on ischemic stroke instead of incorporating all strokes. Ischemic stroke accounts for approximately 87% of all strokes [17] and would have a broad impact on patient outcomes overall. While hemorrhagic stroke shares important risk factors with ischemic stroke such as advanced age and hypertension, hemorrhagic stroke has unique risk factors (such as coagulopathy), underlying pathomechanisms, and associated complications, which require different therapeutic approaches and contribute to an overall greater mortality as compared to ischemic stroke. Excluding hemorrhagic strokes allows for more precise modeling and tailored clinical decision support for acute ischemic stroke care.

In summary, this study addresses existing gaps in predictive analytics for in-hospital mortality focusing on ischemic stroke patients through (a) a pioneering study for developing accurate mortality prediction models tailored to ischemic stroke, which could specifically inform therapeutic decisions for optimal treatment and support resource allocation such as intensive monitoring or withdrawal of maximum therapy; (b) leveraging comprehensive variables in the MIMIC-IV datasets as well as their dynamics through sliding windows; (c) conducting model fairness analyses to understand potential biases for demographically disadvantaged populations to inform future model implementation.

## Methods

### Experimental design

**Fig. 1.**
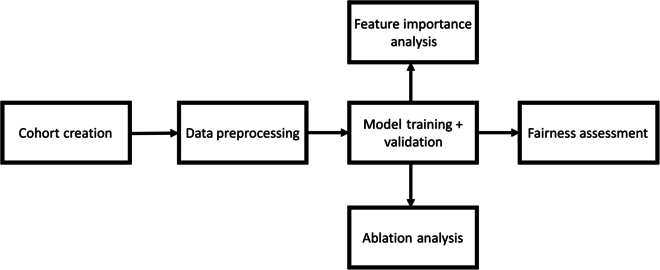
System workflow overview.

We formulated the mortality prediction into a binary classification problem, and the overall system workflow is depicted in Fig. 1. We first created our cohort from the MIMIC-IV dataset and then performed data preprocessing (including feature extraction) before model training and validation. Finally, various analyses were conducted to assess individual features’ importance (feature importance analysis), group features’ contribution (ablation analysis), and the model’s fairness.

### Data sources and cohort creation

**Fig. 2.**
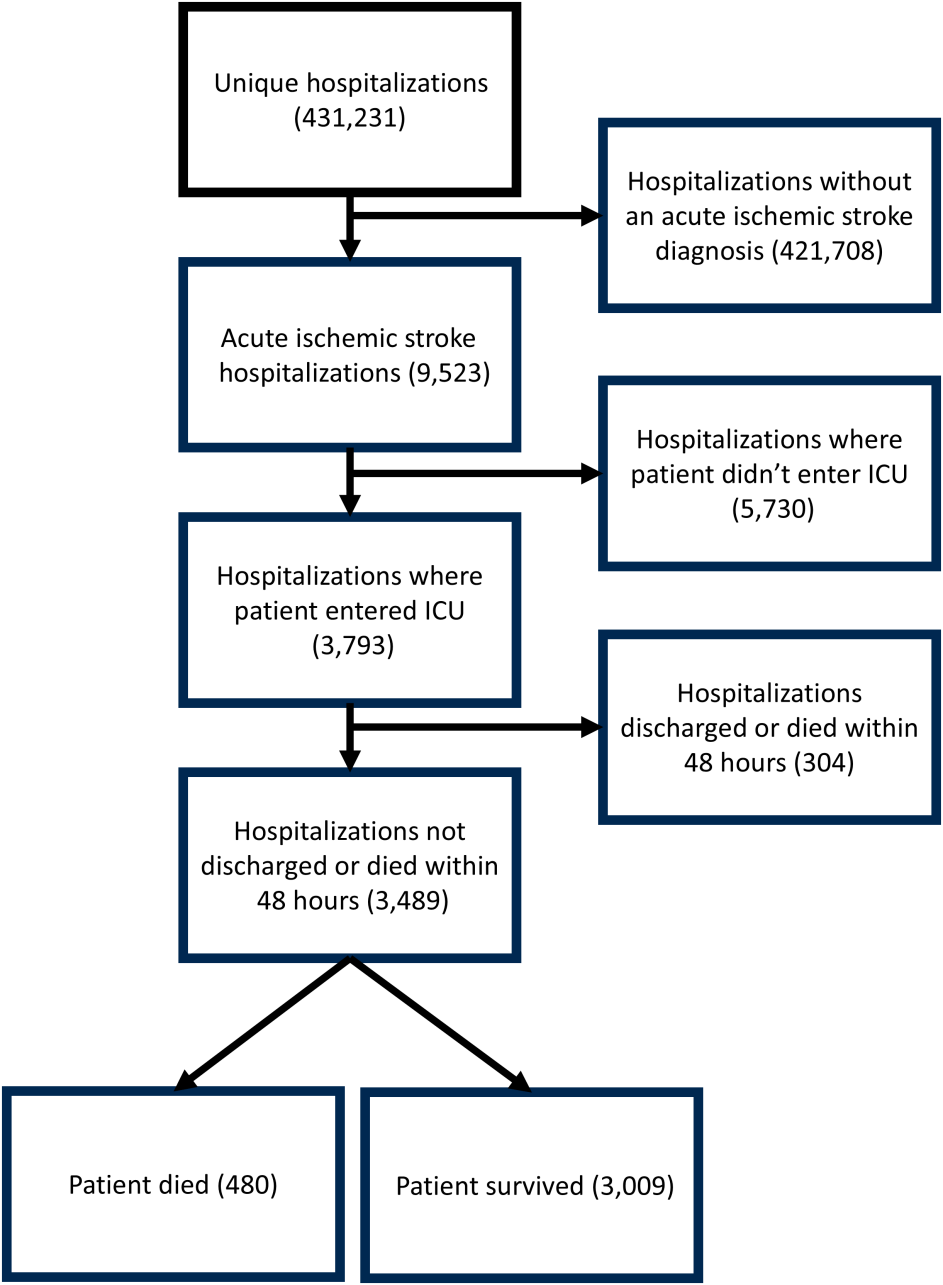
Flow chart of cohort creation.

The MIMIC-IV database provides critical care electronic health record data on 431,231 hospitalizations from 2008 to 2019 for 299,712 patients admitted to the ICU at Beth Israel Deaconess Medical Center in Boston, MA [12]. We selected patients who had at least one diagnostic code for acute ischemic stroke (ICD 10 I63.* or ICD 9 beginning with 433, 434, or 436). Recorded vital sign data in MIMIC-IV are only available from patients who entered the ICU, so hospitalizations in which the patient was not admitted to the ICU were excluded. Only the first ICU admission was considered for patients who required multiple ICU admissions during a single hospitalization. Finally, 304 patients with less than a minimum of 48 h of data were excluded (of these, 104 died). These patients may have limited information of value available in the record due to discharge or transfer, or potentially inconsistent input features with more extreme values. After all exclusions, 3,489 hospitalizations remained for analysis, as shown in Fig. 2. Of these, 3,009 patients survived in the hospital, whereas 480 died before discharge (13.8% mortality). All data were deidentified and publicly available. The corresponding data user agreement was fully executed.

### Data preprocessing and feature engineering

Each type of data provided by MIMIC-IV was processed separately (Table S1). Based on prior related studies and human domain experts, we used demographic, hospitalization information, prescription, laboratory results, vital signs, input (intake), output, and procedure data from the first 48 h of the hospitalization. A 48-h window was chosen because it was found optimal in a previous study using the MIMIC-IV dataset to predict stroke mortality [18]. Demographic data included age as a continuous variable, and categorical data on sex, language, race, and marital status were represented numerically using one-hot encoding. Hospitalization information, including admission type (i.e., urgent and surgical same-day admission) and location (i.e., emergency room and transfer from the hospital), was also one-hot encoded. For all patients, medication use was included if the patient had at least one prescription or treatment within the first 48 h of admission or before the admission. Summary statistics (i.e., minimum, mean, maximum, standard deviation, initial value, and number of measurements) were derived from the first laboratory results and vital signs recorded upon admission and in 8-h windows, creating 6 (8-h windows within 48 h) total windows. Summary statistics, as well as the overall sum, were derived in daily intervals for oral and intravenous input and output. We did not include the comorbidity data from the diagnosis table because the data are not timestamped. Finally, all variables with >40% missingness rate were removed to make sure meaningful patterns can be learned.

### Model training and validation

We explored the state-of-the-art XGBoost gradient boosting tree model for this task and compared it with several conventional ML algorithms. XGBoost [19] has demonstrated promising performance in previous studies using the MIMIC dataset [9,10,20]. XGBoost’s scale_pos_weight parameter was set to 6.3 (3,009 negative observations/480 positive observations). The tree method parameter was set to “hist” to optimize efficiency, and early stopping rounds were set to 50 for hyperparameter tuning to increase efficiency.

We implemented 2 additional conventional ML models, logistic regression and random forest, to compare their performance with the XGBoost model. We chose those 3 models in this study based on their complementary strengths and experimental performance from prior studies. XGBoost is a gradient-boosted decision tree algorithm with regularization capabilities that is optimized for handling high-dimensional data and imbalanced datasets. Random forests [21] combine multiple decision trees in an ensemble manner to make a prediction, which in our use case is a binary classification. It is known for its ability to handle nonlinear relationships and mitigate overfitting through bagging. Logistic regression [22] is a common statistical method used to predict probabilities of binary outcomes using a logistic curve. Its advantages include simplicity, interpretability, and robustness in linear relationships. The class weight parameter for both conventional ML models was set to “balanced” to account for the imbalanced dataset, and the maximum number of iterations and penalty for logistic regression was set to 200 and L2, respectively. For the XGBoost model, lambda, alpha, max_depth, eta, min_child_weight, subsample, colsample_bytree, gamma, grow_policy, learning_rate, and n_estimators were tuned. For the random forest model, n_estimators, max_depth, min_samples_split, and min_samples_leaf were tuned. For the logistic regression model, the C and solver hyperparameters were tuned.

We randomly split our data into training (80%) and testing (20%) in a stratified manner to ensure similar class distributions in training and testing data. All models were tuned using 10-fold cross-validation on the training data. We tuned all 3 models for F1 score and area under the receiver operating characteristic curve (AUC ROC), respectively, and for each hyperparameter tuning round (based on F1 or AUC ROC), we reported the average accuracy, precision, recall, F1 score, and AUC ROC in the 10 cross-validation setting. After hyperparameter tuning, we retrained a model using the optimal hyperparameters on the entire training dataset and tested it on the testing set by bootstrapping the testing data 1,000 times. We used the bootstrapped models’ results to create 95% confidence intervals.

### Feature importance analysis

We analyzed the feature importance of the XGBoost model tuned for an optimal AUC ROC score. XGBoost assigns each feature a coefficient score, with higher scores indicating more useful features for the model. More useful features typically contribute more during the model’s decision-making process. We identified the top 15 most important features from the best-performing XGBoost model tuned for AUC ROC.

### Fairness assessment

The fairness of the final tuned XGBoost model was evaluated by recording the effectiveness of the model for various sex and racial groups based on AUC ROC, precision, recall, and F1 score. The Fairlearn python library was employed to calculate these metrics for each demographic group [23].

### Ablation analysis

To determine which groups of features were contributing most to the model, we conducted an ablation analysis using the final XGBoost model tuned for AUC ROC. For each iteration, we removed one feature category (e.g., demographic, hospitalization information, medication, procedure, laboratory, vitals/clinical assessments, and input/ingredient/output events) only and compared the resulting performance with the model using all the features. Additionally, a model with only GCS features and a model with every non-GCS feature were created to determine the effect of GCS scores on the model.

All analyses used Python 3.11 with libraries including scikit-learn version 1.3.0 and pandas version 2.0.3. *P* values were generated to compare alive versus deceased in both the training and validation datasets based on demographic variables using the TableOne python library’s 2-way analysis of variance (ANOVA) tests or chi-square test for more than 2 categories, with *P* < 0.05 considered significant [24].

## Results

### Demographic characteristics of training and validation subjects

Splitting the dataset into a training and validation set resulted in 2 randomly created groups with an equal proportion of patients who died in the hospital (13.8%). The age, race, and sex demographics of patients in the validation and training sets are similar (Table 1).

**Table 1.** Demographics of training and validation sets

Demographics	Training (*n* = 2,791)			Validation (*n* = 698)		
	Alive (*n* = 2,407)	Deceased (*n* = 384)	*P* value	Alive (*n* = 602)	Deceased (*n* = 96)	*P* value
Mean age (SD) ^a^	68.1 (15.3)	71.4 (14.0)	<0.001	68.4 (15.2)	72.1 (13.2)	0.013
Sex
Male	*n* = 1,243 (51.6%)	*n* = 188 (49.0%)	0.357	*n* = 297 (49.3%)	*n* = 48 (50.0%)	0.991
Female	*n* = 1,164 (48.4%)	*n* = 196 (51.0%)		*n* = 305 (50.7%)	*n* = 48 (50.0%)	
Race
Non-Hispanic white	*n* = 1,597 (66.3%)	*n* = 217 (56.5%)	<0.001	*n* = 402 (66.8%)	*n* = 53 (55.2%)	0.128
Black	*n* = 239 (9.9%)	*n* = 38 (9.9%)		*n* = 55 (9.1%)	*n* = 12 (12.5%)	
Hispanic	*n* = 83 (3.4%)	*n* = 7 (1.8%)		*n* = 23 (3.8%)	*n* = 2 (2.1%)	
Asian	*n* = 54 (2.2%)	*n* = 14 (3.6%)		*n* = 13 (2.2%)	*n* = 3 (3.1%)	
Other	*n* = 434 (18.0%)	*n* = 108 (28.1%)		*n* = 109 (18.1%)	*n* = 26 (27.1%)	

^a^ All patients over 89 years old are assigned to 91 years old in the MIMIC-IV dataset for privacy.

### Model performance

Results with hyperparameter tuning (10-fold cross-validation on training data) show that XGBoost achieved the best performance of 0.855 for AUC ROC and 0.539 for F1, compared with random forests (0.842 for AUC ROC and 0.516 for F1) and logistic regression (0.808 for AUC ROC and 0.462 for F1). Models with hyperparameter tuning outperform their counterparts without tuning across different models (Table 2).

**Table 2.** Performance of models on the training set. Logistic regression, random forest, and XGBoost results on cross-validation before and after hyperparameter tuning.

Models	Metrics	Performance on training set (10-fold cross-validation)		
		Untuned	Tuned for AUC ROC	Tuned for F1 score
Logistic regression	Accuracy	0.694	0.810	0.808
	Recall	0.726	0.570	0.604
	Precision	0.273	0.374	0.375
	F1 score	0.397	0.451	0.462
	AUC ROC	0.764	0.803	0.813
Random forest	Accuracy	0.870	0.869	0.842
	Recall	0.133	0.271	0.607
	Precision	0.597	0.553	0.450
	F1 score	0.215	0.360	0.516
	AUC ROC	0.857	0.868	0.862
XGBoost	Accuracy	0.871	0.852	0.855
	Recall	0.375	0.586	0.620
	Precision	0.547	0.475	0.480
	F1 score	0.442	0.522	0.539
	AUC ROC	0.862	0.868	0.873

### Bootstrapping-based confidence intervals

We present the results of applying the models tuned for F1 on the testing data (withheld validation data) in Table 3. We also calculated 95% confidence intervals through bootstrapping. We can see that XGBoost demonstrated great generalizability on the testing data, outperforming both random forests and logistic regression across the board. Although logistic regression achieved the lowest performance based on most metrics, it yielded a better recall of 0.562 than random forest (0.532).

**Table 3.** Metrics with confidence intervals for XGBoost, random forest, and logistic regression models after bootstrapping

Models	Tuned for	Accuracy	Recall	Precision	F1 score	AUC ROC
XGBoost	Untuned	0.864 [0.863,0.864]	0.366 [0.363,0.369]	0.509 [0.505,0.513]	0.424 [0.421,0.427]	0.842 [0.841,0.843]
	AUC ROC	0.854 [0.853,0.855]	0.572 [0.569,0.575]	0.473 [0.470,0.476]	0.517 [0.514,0.519]	0.860 [0.858,0.861]
	F1 score	0.853 [0.852,0.854]	0.570 [0.567, 0.573]	0.473 [0.470,0.476]	0.516 [0.513,0.518]	0.859 [0.858,0.860]
Random forest	Untuned	0.868 [0.867,0.868]	0.124 [0.122, 0.126]	0.598 [0.591,0.604]	0.204 [0.201,0.207]	0.828 [0.827,0.829]
	AUC ROC	0.875 [0.874,0.876]	0.249 [0.247, 0.252]	0.614 [0.609,0.619]	0.353 [0.349,0.356]	0.849 [0.848,0.850]
	F1 score	0.838 [0.837,0.839]	0.532 [0.528, 0.535]	0.428 [0.425,0.431]	0.473 [0.470,0.476]	0.846 [0.845,0.847]
Logistic regression	Untuned	0.663 [0.662,0.664]	0.647 [0.644, 0.650]	0.235 [0.233,0.237]	0.344 [0.342,0.346]	0.715 [0.714,0.717]
	AUC ROC	0.778 [0.777,0.779]	0.553 [0.550, 0.556]	0.320 [0.318,0.323]	0.405 [0.402,0.407]	0.739 [0.737,0.741]
	F1 score	0.771 [0.770,0.772]	0.562 [0.558, 0.565]	0.315 [0.312,0.317]	0.402 [0.400,0.405]	0.745 [0.744,0.747]

### AUC ROC curves

**Fig. 3.**
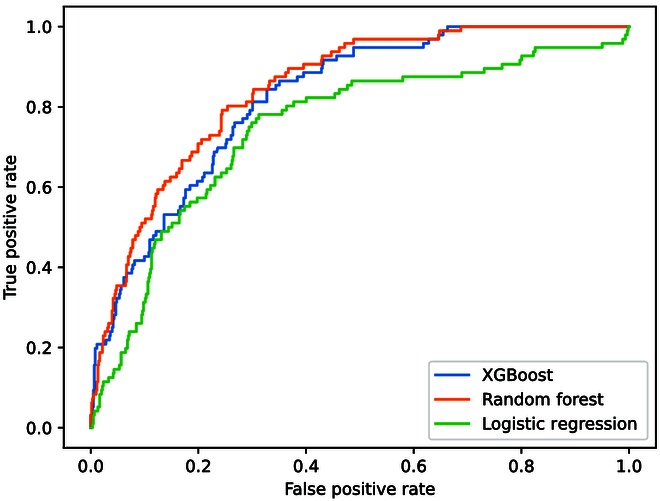
AUC ROC curves of all models using the validation set.

The AUC ROC curves for best-performing models (tuned for F1 score for logistic regression and random forest and tuned for AUC ROC for XGBoost) are presented in Fig. 3. Although there are some overlaps between XGBoost and random forest, XGBoost demonstrated an overall better discriminative ability, especially when the false positive rate is around 0.3 (see Table 3 for other corresponding metrics).

### Model fairness evaluation

We presented the fairness evaluation results for the XGBoost model tuned for AUC ROC in Table 4. Overall, the model’s performance is fair across male and female groups based on both F1 and AUC. Similarly, the model shows fair discriminative power across race/ethnicity groups (AUC). Interestingly, we found that the model performs better (F1 score and AUC) even for less representative racial groups, including non-Hispanic Asian (0.8 F1 score), Black (0.6 F1 score), and Hispanic (0.571 F1 score) compared with the non-Hispanic white reference group (0.504 F1 score). Due to the small sample size for those groups, more investigations are warranted for further validation. Additionally, the model performed worst for individuals with an unspecified race, with an AUC ROC of 0.838 and an F1 score of 0.473.

**Table 4.** Performance of the model by sex and race/ethnicity

Demographics Groups	Recall	Precision	F1 score	AUC ROC
Sex				
Male (*n* = 345)	0.542	0.491	0.515	0.867
Female (*n* = 353)	0.604	0.460	0.523	0.853
Race/ethnicity				
Non-Hispanic white (*n* = 455)	0.547	0.468	0.504	0.852
Black (*n* = 67)	0.750	0.500	0.600	0.880
Hispanic (*n* = 25)	1.000	0.400	0.571	0.913
Asian (*n* = 16)	0.667	1.000	0.800	0.974
Unspecified race (*n* = 135)	0.500	0.448	0.473	0.838

### Feature importances

**Fig. 4.**
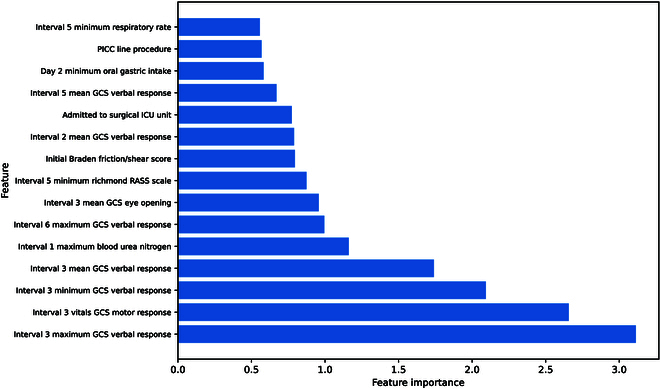
Top 15 important features identified by the XGBoost model tuned for AUC ROC.

The sum of all features’ importance values from the model is 100, so the feature importance represents the percentage of effect a variable had on the model (Fig. 4). The 4 most important features were summary statistics from Interval 3 GCS Verbal and Motor Tests. The rest of the top 15 features included BUN, minimum respiratory rate measurement, and additional GCS and Richmond RASS scale features.

### Ablation analysis

The best-performing XGBoost model tuned for AUC ROC achieved an accuracy of 0.85, an F1 score of 0.52, and an AUC ROC of 0.86 (Table 5). Removing medications did not have a major impact on the model. After removing procedures from the model, the AUC ROC decreased by 0.01, and the F1 score decreased by 0.04. Removing demographic data resulted in the model’s F1 score decreasing by 0.04. Similarly, removing input and output events decreased the F1 score by 0.05. Removing labs from the model slightly decreased the F1 score but increased the AUC. Finally, removing vitals and clinical assessment data from the model had the greatest effect, decreasing the F1 score by 0.03 and the AUC ROC by 0.05.

**Table 5.** Metrics for groups of features using feature ablation analysis (testing data) ^a^

Feature	Accuracy	Recall	Precision	F1 score	AUC ROC
All features	0.854	0.573	0.474	0.519	0.860
Demographics	0.844	0.531	0.444	0.483	0.859
Medications	0.841	0.583	0.441	0.502	0.857
Procedures	0.840	0.542	0.433	0.481	0.851
Input/output	0.841	0.521	0.435	0.474	0.857
Labs	0.845	0.594	0.452	0.514	0.867
Vitals/clinical assessments	0.835	0.583	0.427	0.493	0.814

^a^ Groups of features are removed one at a time to assess their contribution.

As GCS is shown to be one of the top features, another ablation experiment shows that removing GCS features does not lead to noticeably lower performance (Table 6). We found that the model with only GCS features performed worse than the full model, with a 0.04 lower AUC ROC and a 0.08 lower F1 score.

**Table 6.** Metrics for the XGBoost model using combinations of GCS and non-GCS features

Features	Accuracy	Recall	Precision	F1 score	AUC ROC
All features	0.854	0.573	0.474	0.519	0.860
All—GCS	0.844	0.552	0.445	0.493	0.855
Only GCS	0.812	0.531	0.372	0.438	0.822

## Discussion

We describe the development of an XGBoost model to predict acute ischemic stroke mortality during ICU hospitalization using the MIMIC-IV dataset. XGBoost outperformed (see Table 3) traditional ML models (i.e., logistic regression and random forest) based on both F1 score and AUC ROC. While random forest achieved a similar AUC ROC to XGBoost, the F1 score was much lower because of its poor precision or recall. Compared with logistic regression, random forest tends to yield lower recall and higher precision but achieves much better overall discrimination ability based on AUC ROC. Overall, the XGBoost model likely exhibits advantages, consistent with previous studies (e.g., [25]), over the other models through learning complex nonlinear relationships, employing regularization to prevent overfitting, and better opportunities for fine-tuning. Also notable is that the final tuned XGBoost models’ characteristics suggest that the predictive ability is better for Asian, Black, and Hispanic individuals than for non-Hispanic white individuals. However, small group sample sizes reduce the ability to conclude this.

Previous studies have measured the accuracy of common stroke measures (OASIS, NIHSS, and GCS) to predict stroke mortality. In comparison to those studies, our AUC ROC for the XGBoost model tuned for AUC ROC was higher (0.86). First, one study that used the OASIS score to predict stroke mortality found an AUC ROC of 0.73, analyzing 885 ischemic stroke patients from the MIMIC-III dataset [20]. Second, in a study with 80% ischemic stroke patients and 20% hemorrhagic stroke patients and a similar in-hospital mortality rate (11.7%), GCS achieved an AUC ROC of 0.78 [26]. Third, a model using both an NIHSS score and age had an AUC ROC of 0.83 [27]. However, it is important to note that the comparison is not perfect because these studies used conventional statistical analysis and not ML. These studies did not report on F1 scores or any comparable metrics useful for imbalanced datasets. Thus, it is possible that these scores made predictions heavily weighted toward the majority class despite high AUC ROC scores. Overall, the full model appears more effective than a model using only GCS because the GCS-only model achieved substantially lower accuracy, F1 score, and AUC ROC using only GCS features. In situations where GCS is not available, the model can still perform well reliably.

We identified 15 features that contributed the most to the XGBoost model, where many have been found in other prediction models as well. Previous studies have demonstrated that GCS is associated with stroke mortality [28–30]. Therefore, it is not surprising that GCS-related features were among the top features our model identified. The GCS is a neurological scale that aims to assess the extent of impaired consciousness in all types of acute medical and trauma patients [5], and lower GCS scores indicate a reduced level of consciousness and potentially severe brain injuries. Low GCS levels often occur in patients with severe systemic illnesses and at higher risk of life-threatening complications, thus leading to increased mortality. Furthermore, interval 3 GCS scores were prominent features, consistent with other studies demonstrating that early data during the first 24 h of an ICU admission have more missing data and other challenges [31]. In addition to GCS, lack of responsiveness measured by the Richmond RASS score and altered respiratory rate might be associated with increased in-hospital mortality, whereas healthier patients may be expected to have greater oral dietary intake. Further, the Braden Scale includes a number of subscales (e.g., sensory perception, activity, and mobility) that likely overlap with other scales (such as GCS), where unresponsiveness and inability to change body position or perceive discomfort are generally poor prognostic indicators.

Another notable feature identified through importance analysis is BUN, the primary end-product of protein metabolism that is renally excreted. A higher BUN level reflects excessive protein breakdown (hypercatabolic state) or when renal function decreases. It is a commonly found repeated measure in blood chemistries in the ICU and generally found to predict mortality overall [32] in critically ill patients, including those with acute coronary syndrome and heart failure [33–36]. In acute stroke, there are several mechanisms that may contribute to elevated BUN levels: a hypercatabolic state during the acute phase of stroke, dehydration and poor renal perfusion, and an inflammatory response to stroke. Explanations for the increased mortality risk in acute stroke have included hemodynamic changes causing poor renal perfusion [33], sympathetic nervous activation associated with urea reabsorption and renal dysfunction [37], and cerebral microbleeding associated with chronic renal failure [38]. Our study identified the BUN level in interval 1 as a key feature, emphasizing its importance in early prediction of mortality that may reflect a number of different and complex physiologic pathways. Its importance in longer-term prediction has also been recognized [39].

There were several limitations. First, the mortality outcome relies on the death record in the hospital, and it is likely that if a patient was made CMO (comfort measures only), they were discharged to hospice. Second, this study only included patients admitted to the ICU for at least 48 h. Excluding patients with less than 48 h of available data could affect the model’s representativeness. However, excluding those patients addresses potential sparsity of data, allows for consideration of more time-dependent variables, and reflects possible stabilization that may occur shortly after admission. It also excludes patients that were admitted to the ICU solely based on local protocols (such as post-thrombolysis monitoring) rather than driven by their disease severity. Third, the model was trained using a single-center dataset, which may reduce generalizability. However, the MIMIC-IV dataset (and its previous version, MIMIC-III) represents a large and diverse cohort of ICU patients. MIMIC has provided a strong foundation for academic and industrial research and has been widely used for model development and validation, including ICU mortality prediction [40–43]. Some studies have shown that external validation of MIMIC-III/MIMIC-IV models on other similar datasets, including eICU data [15] and local health organization data, has demonstrated strong generalizability for blood lactate change prediction [44], mortality prediction [45], and intubation prediction [46]. In our study, we have employed techniques such as cross-validation, withholding separate testing data, and robust feature engineering to mitigate the potential risk of overfitting. Also, we used bootstrapping to calculate confidence intervals and address instability in model performance estimates. In the future, to further evaluate our model’s generalizability, we will conduct an external validation and involve ICU datasets from multiple institutions, e.g., the eICU dataset [15]. We will validate the model’s generalizability in both performance and fairness. Fourth, while some of the factors, such as GCS features, are likely to remain predictive factors in most settings, some factors may be reflective of the hospital’s protocols and local care provided. Therefore, model accuracy may substantially vary across hospitals and time periods. A similar claim was made for sepsis model algorithms in practice that performed variably among different hospitals [47]. Finally, and importantly, our observations on model performance differences across ethnic/racial groups need to be tempered by the small sample sizes of the non-white groups.

## Conclusion

We have developed and validated ML models for mortality prediction among acute ischemic stroke patients in the ICU setting. Our analyses using the MIMIC-IV dataset have shown that XGBoost demonstrated promising results in both performance and fairness. Once externally validated on geographically and demographically diverse populations, it has the potential to provide better clinical decision support by proactively identifying patients at high risk for mortality.

## Ethical Approval

MIMIC-IV is a publicly available data. The collection of patient information and creation of the research resource for MIMIC-IV was reviewed by the Institutional Review Board at the Beth Israel Deaconess Medical Center, which granted a waiver of informed consent and approved the data-sharing initiative.

## Data Availability

MIMIC-IV data are publicly available (https://physionet.org/content/mimiciv/3.1/) with minimal steps to become credentialed.
