# Supplementary material for: In-Hospital Mortality Prediction among Intensive Care Unit Patients with Acute Ischemic Stroke: A Machine Learning Approach
Source: Health Data Sci. 2025 Mar 17;5:0179. doi: 10.34133/hds.0179 (PMC11912875; doi:10.34133/hds.0179)
Supplement: Supplementary 1 — Table S1 [file hds.0179.f1.docx]

**Supplementary Materials**

|  | **Demographic/Admission** | **Prescription** | **Lab/ Vital** | **Input/ Output** | **Procedure** |
| --- | --- | --- | --- | --- | --- |
| Binary (true or false) | X | X |  |  | X |
| Summary statistics (initial, minimum, mean, maximum, standard deviation, and other numeric measurements) |  |  | X | X |  |
| Sum of all measurements |  |  |  | X |  |
| Recorded in 8-hour intervals |  |  | X |  |  |
| Recorded in 24-hour intervals |  |  |  | X |  |

**Supplementary Table 1***.* Preprocessing techniques for various types of data.
